# Supplementary material for: Where are you hiding the pangolins? screening tools to detect illicit contraband at international borders and their adaptability for illegal wildlife trafficking
Source: PLoS One. 2024 Apr 3;19(4):e0299152. doi: 10.1371/journal.pone.0299152 (PMC10990205; doi:10.1371/journal.pone.0299152)
Supplement: S1 Table — (DOCX) [file pone.0299152.s002.docx]

**Table S1. Search strategy for wildlife detection tools.**

|  | | | **Database** | | | | | | |
| --- | --- | --- | --- | --- | --- | --- | --- | --- | --- |
|  |  |  | **PubMed** | **CAB Abstracts** | | **Web of Science** | **Google Scholar** | **IEEE Xplore** | **Scopus** |
| ("illegal wildlife trade" OR "wildlife trade" OR "wildlife traffic*" OR “wildlife smuggl*”) | **AND** | (postal OR transport OR traffic* OR airport OR freight OR mail OR truck) AND (detect* OR surveillance) | 25 | 8 | | 38 | 5,200 | 0 | 50 |
|  | **AND** | (detect* OR surveillance) | 100 | 45 | | 137 | 8,410 | 1 | 205 |
|  | **AND** | (DNA OR "detect* dogs" OR "sniffer dogs" OR x-ray OR xray OR CT OR fluor* OR "smart shipping" OR thermo* OR isotop* OR "mass spectro*" OR "chromato*" OR “air sampl*”) | 73 | 29 | | 99 | 1,050 | 0 | 144 |
|  | **AND** | (x-ray OR "detection dog" OR dog OR isotop* OR scan* OR fluoro* OR DNA OR "electric nose" OR document*) AND (transport OR ship* OR mail OR postal OR airport OR truck OR freight) | 14 | 4 | | 22 | 582 | 0 | 32 |
|  | **AND** | (mail OR post* OR ship*) | 18 | 5 | | 43 | 11,500 | 2 | 124 |
|  | **AND** | (‘sniffer dog’ OR ‘detect* dog’ OR xray OR CT OR ‘radiation’ OR fluoroscopy OR scan* OR AI OR ‘artificial intelligence’ OR ‘air sampl*’ OR isotop* OR ‘thermal imaging’) | 29 | 10 | | 58 | 4,130 | 0 | 0 |
|  |  | (illegal AND wildlife AND trade AND detect*) | 60 | 48 | | 143 | 34,700 | 2 | 131 |
|  |  |  |  | |  |  |  | **TOTAL** | 67,271 |
