# Supplementary material for: Where are you hiding the pangolins? screening tools to detect illicit contraband at international borders and their adaptability for illegal wildlife trafficking
Source: PLoS One. 2024 Apr 3;19(4):e0299152. doi: 10.1371/journal.pone.0299152 (PMC10990205; doi:10.1371/journal.pone.0299152)
Supplement: S2 Table — (DOCX) [file pone.0299152.s003.docx]

|  | | | | |  |  | **Database** | | | | |
| --- | --- | --- | --- | --- | --- | --- | --- | --- | --- | --- | --- |
|  | | | | |  |  | **PubMed** | **CAB Abstracts** | **Web of Science** | **IEEE Xplore** | **Scopus** |
| ("drug smuggl*" OR narcotic*) | **AND** | (detection OR screening OR scanning) | **AND** | (traffic* OR postal OR ship* OR mail OR airport OR cargo OR luggage OR parcel) | | | 410 | 2 | 119 | 17 | 345 |
| weapon | **AND** | detection | **AND** | (airport* OR cargo OR ship*) | | | 34 | 2 | 202 | 145 | 343 |
| smuggl* | **AND** | (detect* OR screening OR scanning) | **AND** | (traffic* OR postal OR ship* OR mail OR airport OR cargo OR luggage OR parcel) | | | 75 | 8 | 198 | 63 | 322 |
| (illicit OR illegal OR contraband) | **AND** | (detect* OR screening) | **AND** | (postal OR ship* OR mail OR airport OR cargo OR luggage OR parcel) | | | 316 | 30 | 681 | 285 | 900 |
|  |  |  |  |  | | |  |  |  | **TOTAL** | 4,497 |

**Table S2. Search strategy for screening tools for illicit contraband detection.**
