# Supplementary material for: Where are you hiding the pangolins? screening tools to detect illicit contraband at international borders and their adaptability for illegal wildlife trafficking
Source: PLoS One. 2024 Apr 3;19(4):e0299152. doi: 10.1371/journal.pone.0299152 (PMC10990205; doi:10.1371/journal.pone.0299152)
Supplement: S3 Table — Detection tools described in the literature which primarily utilise ionising radiation. (DOCX) [file pone.0299152.s004.docx]

**Table S3. Ionising radiation detection tools.** Detection tools described in the literature which primarily utilise ionising radiation.

| **Inspection system** | **Description** |
| --- | --- |
| **High energy  [transmission] x-ray** | High-energy transmission x-rays are powerful imaging tools used in contraband detection. These x-rays can penetrate dense materials, including steel, and provide detailed scans as they pass through an object to produce a high-resolution 2D image, making them essential for uncovering hidden contraband, weapons, or illicit items in cargo, luggage, or vehicles. Imaging processing methods are employed for material discrimination. |
| **Mobile cargo container inspection unit (medium energy x-ray)** | A mobile cargo container inspection unit is a portable system equipped with advanced scanning technology, such as x-rays (450kVp medium-energy x-ray generator) and photodiode-based detectors, designed for contraband detection in shipping containers. It efficiently screens large volumes of cargo for hidden contraband, including drugs and illegal goods. |
| **Continuous wave x-ray** | Continuous wave x-ray technology is used for contraband screening by emitting a continuous x-ray beam through an object. It measures changes in the beam's intensity caused by variations in material density, producing a 3D image which can help to localise threats in regions of interest and reveal concealed items. This non-invasive method is effective for detecting contraband in luggage, parcels, and cargo. The system can also discriminate and identify fissionable materials, specific chemical isotopes, and other materials based on atomic number and density. |
| **Dual-energy x-ray** | Dual-energy x-ray is a contraband screening technique that utilizes two x-ray energy levels to distinguish materials. It enhances the detection of contraband by differentiating between organic and inorganic substances, making it effective for identifying concealed items like drugs, explosives, and weapons in luggage, cargo, and baggage screening applications. |
| **Dual-angle x-ray** | Dual-angle x-ray, also known as multi-view X-ray scanning, employs multiple x-ray angles to examine objects from different perspectives. This method enhances contraband screening by providing a more comprehensive view of items, therefore ensuring high speed, continuous screening. |
| **Energy dispersive x-ray diffraction** | Energy dispersive x-ray diffraction is a technique used in contraband screening to analyze the elemental composition of materials. By measuring the scattered c-rays, it identifies substances and can detect concealed contraband like drugs, explosives, and weapons. This method enhances security by providing insight into the chemical composition of scanned objects. This has also been demonstrated in combination with the multivariate analysis technique of partial least squares regression for drug identification. |
| **X-ray backscatter imaging** | X-ray backscatter imaging reflects x-rays off an object to create an image of its concealed contents. It's used for identifying hidden contraband like drugs and weapons. The lobster-eye objective is used to detect x-ray backscatter and improve image resolution. Organic and inorganic materials can be distinguished based on the intensity of x-ray scattering. |
| **Gamma radiation** | Gamma radiation is employed in contraband screening to detect hidden materials and contraband items in cargo and luggage. It utilizes high-energy gamma rays to penetrate objects and measure the resulting radiation signatures (photoelectric effect and Comptom scattering). This method is effective in identifying substances like drugs, explosives, and illicit goods and can distinguish between high- and low-density materials. |
| **Gamma resonance technology (GRT)** | GRT is a contraband screening method that uses gamma rays to identify the elemental composition of materials. It relies on the identification of elemental composition, particularly nitrogen, but also oxygen, chlorine and other elements of interest. By analyzing the resonant gamma energy levels of specific elements, it can detect concealed contraband, such as drugs and explosives. |
| **Computed Tomography (CT)** | CT is used in contraband screening to create detailed 3D cross-sectional images of objects. It's effective for detecting concealed contraband items, like drugs and weapons, by providing three-dimensional insights into the contents of luggage, cargo, and containers. Multi-Detector Computed Tomography (MDCT) uses multiple x-ray detectors to create detailed 3D images for contraband screening. Megavolt Computed Tomography (MVCT) uses high-energy x-rays to create detailed images for screening. |
| **Passive radiation detection** | Passive radiation detection monitors natural radiation emitted by materials and identifies abnormal radiation levels indicating the presence of radioactive contraband. It can even detect the naturally occurring radioactive isotope K-40 (concentrated in plant leaves due to its role in photosynthesis) emissions from plant contraband. |
