# Supplementary material for: Where are you hiding the pangolins? screening tools to detect illicit contraband at international borders and their adaptability for illegal wildlife trafficking
Source: PLoS One. 2024 Apr 3;19(4):e0299152. doi: 10.1371/journal.pone.0299152 (PMC10990205; doi:10.1371/journal.pone.0299152)
Supplement: S4 Table — Detection tools described in the literature which primarily utilise photon and neutron interrogation techniques. (DOCX) [file pone.0299152.s005.docx]

**Table S4.** **Photon and neutron detection tools.** Detection tools described in the literature which primarily utilise photon and neutron interrogation techniques.

| **Inspection system** | **Description** |
| --- | --- |
| **Tagged neutron inspection system (TNIS/TNM/TNA)** | Tagged neutron inspection systems use 14 MeV neutron sources to bombard inspected objects, causing unique emissions when encountering specific materials like explosives or narcotics. These emissions are then analyzed to determine the elemental composition of a material and therefore enable contraband identification. |
| **Pulsed Fast Thermal Neutron Analysis (PFTNA)** | PFTNA involves emitting short bursts of fast neutrons onto objects, creating thermal neutrons upon interaction with certain materials. The combination of fast inelastic neutron scattering and thermal neutron capture enables elements contained within an object to be measured in a continuous mode. Gamma ray spectra produced by nuclear reactions are analysed and used to determine the elemental composition of an object. |
| **Pulsed Fast-Neutron Analysis (PFNA)** | PFNA employs short bursts of fast monoenergetic neutrons to interact with and analyze materials, identifying hidden substances like explosives or narcotics based on their unique elemental composition. The signatures obtained provide an indication of the elemental composition of a material, where the presence of illicit substances (i.e. cocaine) may be based on the ratio of carbon and oxygen. |
| **Fast-neutron resonance radiography (FNRR) [originally named PFNTS]** | FNRR is a cutting-edge method for contraband detection. It uses fast neutrons to create detailed images of objects, revealing hidden materials like explosives or drugs. FNRR considers the in-depth elemental composition of samples and exploits the characteristic cross-section structures (resonances) of isotopes within the energy range of 1-10 MeV. The object of interest is irradiated with neutrons of a broad spectral distribution within this energy range. By analyzing resonance peaks in the neutron spectrum, FNRR can identify specific substances, enhancing security measures in diverse applications such as airport screening and customs inspections. |
| **Fast neutron activation analysis (FNAA)** | FNAA is a technique used for contraband detection through determining the quantity and composition of elements in a sample. It involves irradiating objects with fast neutrons, leading to the activation of certain elements. By measuring resulting gamma rays, FNAA identifies the elemental composition of materials, helping detect hidden contraband like explosives or narcotics in security and customs inspections. |
| **Neutron backscatter detection** | Neutron backscatter detection is an inelastic neutron scattering technique which uses low-energy neutrons to scan objects. It measures the backscattered neutrons, which interact differently with various materials. Significant neutron backscatter is observed from materials with a large hydrogen content, such as narcotics. As neutrons and gamma rays have quantitatively different backscatter signatures, these signals can be combined complementary and when analysed simultaneously the two independent signatures help to uncover concealed objects. |
| **Integrated photon and neutron radiography** | Integrated photon and neutron radiography is an advanced contraband detection method that combines x-rays with neutrons to provide comprehensive imaging. It offers superior identification capabilities for hidden substances like explosives or drugs by exploiting the complementary information from both photon and neutron interactions, where photons have a higher sensitivity to high-Z materials and neutrons are more sensitive to low-Z materials. |
| **Fast neutron scattering analysis (FNSA)** | Fast neutron scattering analysis employs fast neutrons to interact with materials and analyzes the resulting scattered neutrons. The scattering of a beam of fast monoenergetic neutrons is used to evaluate the elemental composition of a sample, with a particular focus on the concentrations of the elements C, N, O, H (the main constituents of explosives and narcotics). Characteristic scattering signatures for different elements are derived based on time-of-flight and pulse height measurements, then used to identify a material. |
| **Fast neutron transmission spectroscopy (FNTS)** | Fast neutron transmission spectroscopy is a contraband detection method using fast neutrons to scan objects. It measures the transmission of these neutrons through materials and analyzes their energy spectra. Time-of-flight techniques are used to measure the energy spectrum of emitted neutrons from a collimated continuum source, both before and after transmission through an object (i.e. luggage/cargo). Elemental areal densities can be determined and used to determine the presence of contraband. |
| **Fast neutron and gamma radiography (FNGR)** | Fast neutron and gamma radiography is a dual-mode imaging technique. It combines fast neutrons and gamma rays to penetrate cargo containers and provide rapid high-resolution images detailing material composition. It creates 2D images based on density and material composition and can distinguish between inorganic and organic materials. |
| **Fast neutron and x-ray cargo scanner** | Fast neutron and x-ray scanners are advanced security systems used for contraband detection in cargo containers and vehicles. By combining fast neutrons and x-rays, they provide detailed imaging and material composition and identification capabilities. |
