# Supplementary material for: Where are you hiding the pangolins? screening tools to detect illicit contraband at international borders and their adaptability for illegal wildlife trafficking
Source: PLoS One. 2024 Apr 3;19(4):e0299152. doi: 10.1371/journal.pone.0299152 (PMC10990205; doi:10.1371/journal.pone.0299152)
Supplement: S5 Table — Detection tools described in the literature which primarily utilise nuclear material detection systems. (DOCX) [file pone.0299152.s006.docx]

**Table S5. Nuclear detection tools.** Detection tools described in the literature which primarily utilise nuclear material detection systems.

| **Inspection system** | **Description** |
| --- | --- |
| **Photoneutron induced gamma analysis / Photoneutron based prompt gamma-ray neutron activation  analysis (PGNAA)** | Photoneutron induced gamma analysis uses high-energy photons to induce neutron emissions, which interact with materials. The concurrent use of bremsstrahlung x-ray imaging and photoneutron induced gamma ray analysis (with Compton scattering) is used to derive both images and elemental information (Cl, H, Fe, N) to demonstrate the detection of concealed narcotics and explosives. |
| **Photofission system** | A photofission system utilizes high-energy photons to induce fission reactions. By detecting the resultant nuclear reactions (i.e. detection of delayed neutrons and gamma rays following photofission), it can identify hidden contraband such as nuclear materials or explosives. |
| **Cosmic ray muon tomography** | Cosmic ray muon tomography is used to generate 3D images of cargo containers based on the Coulomb scattering of the muons to detect high atomic number (high-Z) materials. This method is particularly useful for investigating high density (high-Z) materials, where the amount of scattering is proportional to the Z^2^ of the material. Muons are more deeply penetrating than x-rays and can penetrate shielded special nuclear material (SNM) with minimal absorption. |
| **The nuclear car wash scanning system** | The nuclear car wash scanning system employs a combination of gamma-ray detectors, neutron detectors, and radiation imaging systems to detect the beta-delayed high energy neutrons and beta-delayed high-energy gamma-radiation produced. It employs a combination of radiation sources and detectors to scan vehicles quickly and non-invasively. |
| **Nuclear quadrupole resonance (NQR)** | NQR is a specialized technique which identifies specific compounds by detecting the unique electromagnetic signals emitted by their quadrupolar nuclei when subjected to radiofrequency pulses. NQR is especially effective for detecting explosives and certain narcotics. Interpretation of NQR signals can be optimised through the application of detection algorithms, artificial intelligence and deep learning. |
| **Nuclear resonance fluorescence (NRF)** | NRF involves bombarding materials with photons to excite their nuclei, which emit characteristic gamma rays. By analyzing these gamma rays, NRF can identify hidden contraband like explosives or nuclear materials. |
| **Low energy nuclear reaction imaging** | Low energy nuclear reaction imaging uses low-energy nuclear reactions to create images of objects. This technique could be used to identify the presence of shielded special nuclear material. This system uses a dual-particle approach, where the photons are used to locate high-atomic nuclear materials, whereas the high-energy photons and neutrons confirm the presence of fissionable material. |
| **Pulsed Photonuclear Assessment (PPA) inspection system** | The PPA inspection system utilises high-energy photons to induce nuclear reactions within materials (i.e. shielded nuclear materials), producing unique signatures. |
