# Supplementary material for: Where are you hiding the pangolins? screening tools to detect illicit contraband at international borders and their adaptability for illegal wildlife trafficking
Source: PLoS One. 2024 Apr 3;19(4):e0299152. doi: 10.1371/journal.pone.0299152 (PMC10990205; doi:10.1371/journal.pone.0299152)
Supplement: S6 Table — Detection tools described in the literature which primarily utilise non-ionising radiation. (DOCX) [file pone.0299152.s007.docx]

**Table S6. Non-ionising detection tools.** Detection tools described in the literature which primarily utilise non-ionising radiation.

| **Inspection system** | **Description** |
| --- | --- |
| **Active millimeter wave imaging** | Active millimeter-wave imaging is a whole-body imaging technology. It emits low-power millimeter waves and measures their reflections to create high-resolution 3D images. This technique can reveal concealed items, such as weapons or drugs, on a person's body, shoes, or within objects. |
| **Passive millimeter wave imaging** | Passive millimeter-wave imaging is a contraband detection method that captures naturally emitted millimeter-wave radiation from objects and individuals. It produces detailed images highlighting hidden items, such as weapons or explosives, without emitting radiation itself. Image fusion technology (combining a visual image with a millimeter-wave image) can improve detection efficiency. |
| **Ultra-wide band 3D microwave imaging scanner** | The ultra-wide band 3D microwave imaging scanner is a contraband detection system which utilizes short-duration, high-frequency microwave pulses to create detailed 3D images of objects or people, revealing concealed items. Automated target recognition software (SIFT) can aid in the detection and classification of 3D objects. |
| **High frequency ground penetrating radar** | High-frequency ground-penetrating radar (GPR) emits radio waves into the ground or objects and measures the reflected signals to create detailed subsurface images. Reflection hyperbola or other anomalies may be visualised where contraband is hidden. |
| **Terahertz spectrometry** | Terahertz spectrometry uses terahertz radiation to analyze the unique spectral signatures of objects or materials. Passive terahertz systems utilise naturally emitted radiation by the human body to detect hidden objects (i.e. weapons hidden under clothing). |
| **Infrared imaging (IR)** | Infrared imaging captures thermal radiation emitted by the body or objects to form an image. By identifying variations in temperature, it can reveal hidden items like weapons or drugs either on individuals or within objects. The relative temperature of [concealed] objects and the human body are measured. |
| **Short-wave infrared imaging** | Short-wave infrared uses electromagnetic radiation within a subset of the infrared band and is based on vibrational overtones. Photons are reflected or absorbed by the object and interpreted to produce high-resolution images. |
