# Supplementary material for: Where are you hiding the pangolins? screening tools to detect illicit contraband at international borders and their adaptability for illegal wildlife trafficking
Source: PLoS One. 2024 Apr 3;19(4):e0299152. doi: 10.1371/journal.pone.0299152 (PMC10990205; doi:10.1371/journal.pone.0299152)
Supplement: S7 Table — Detection tools described in the literature which primarily utilise trace detection techniques. (DOCX) [file pone.0299152.s008.docx]

**Table S7.** **Trace detection techniques.** Detection tools described in the literature which primarily utilise trace detection techniques.

| **Inspection system** | **Description** |
| --- | --- |
| **Electronic nose (eNose) and volatile organic compound (VOC)  detection** | The electronic nose is a contraband detection device that mimics olfaction. It uses sensors to detect and analyze chemical odours emitted by substances like explosives or drugs. The detector is composed of a sampler and an analyser, to first extract the vapours then analyse them. |
| **The surface acoustic wave and gas chromatograph (SAW/GC) system** | The SAW/GC system combines surface acoustic wave sensors with gas chromatography to detect and analyze chemical compounds associated with contraband, such as explosives or drugs. GC separates the organic compounds into pure components, meanwhile SAW is used for quantitative analysis. |
| **Single-particle aerosol mass spectrometry  system (SPAMS)** | SPAMS analyzes individual airborne particles or aerosols generated by CBRN&E materials by time-of-flight mass spectrometry to identify substances. |
| **Aerodynamic assisted thermodesorption mass spectrometry (AATD-MS)** | AATD-MS utilizes aerodynamic principles to release and analyze volatile compounds from objects. Molecules present in a sample are identified based on their mass-to-charge ratio. |
| **Atmospheric flow tube-mass spectrometry (AFT-MS)** | AFT-MS uses controlled atmospheric flow to introduce and analyze substances. The signal intensities obtained are compared with the standard curve previously generated for that compound to quantify the sample. The vapour concentration is determined in accordance with the flow and collection time. |
| **Ion mobility spectrometry (IMS)** | IMS is a contraband detection method that ionizes substances and measures their mobility in a drift tube. It identifies specific compounds by their characteristic drift times, making it valuable for detecting hidden contraband such as explosives or narcotics. The technology is widely used for the detection of nitro-organic explosives in luggage. |
| **Paper spray mass spectrometry (PS-MS)** | PS-MS involves applying a sample to paper, ionizing it, and then analyzing the resulting ions using mass spectrometry. This method rapidly identifies substances like drugs or explosives. Solid and liquid samples can both be analysed. The paper itself can be modified chemically or physically to enhance analyte detection. |
| **Direct analysis in real time mass spectrometry  (DART-MS)** | DART-MS ionizes compounds from surfaces and analyzes their mass spectrometry signatures to rapidly identifies substances. |
| **Solid phase microextraction with explosive and ion mobility spectrometry (SPME-IMS)** | SPME-IMS employs a microextraction technique to capture and analyze volatile compounds from objects. SPME has been coupled with IMS as a sample pre-concentration device to improve the detection of concealed explosives. |
| **Combined molecular property spectrometer (MPS) and gamma spectrometer** | The combined MPS and gamma spectrometer combines molecular analysis with gamma spectroscopy to identify and characterize substances based on their properties and radiation emissions. These two subsystems combined enable enhanced threat detection in cargo containers. |
| **Container inspection system:  Chemical (hand-held ion mobility spectrometer) and radiological (dual scintillation counter)** | The Container Inspection System combines a hand-held ion mobility spectrometer for chemical analysis and a dual scintillation counter for radiological screening. This integrated approach enables the detection of both chemical and radiological contraband, such as explosives or nuclear materials. |
| **Detector dogs** | Detection dogs are highly trained canines used to identify contraband, such as drugs, explosives, or illicit substances. Their exceptional sense of smell enables them to detect hidden items with remarkable accuracy. |
| **Thermal desorption electrospray ionisation mass spectrometry  (TD-ESI/MS)** | TD-ESI/MS heats samples to release volatile compounds, which are then ionized and analyzed using mass spectrometry. This method rapidly identifies substances like explosives or narcotics. |
| **Laser-induced immunofluorometric biosensor** | A laser-induced immunofluorometric biosensor uses laser-induced fluorescence to detect specific antibodies, antigens, or biomolecules associated with contraband substances. In the presence of the target compound (i.e. cocaine), antibody binding sites will be blocked, therefore there will be a breakthrough of the labelled protein detectable by laser induced fluorescence. |
| **Continuous flow immunosensor** | A continuous flow immunosensor relies on antibody-antigen binding. It employs antibodies to detect specific substances like drugs or explosives in a continuous flowing sample, where the amount of displacement of the fluorescent analyte is proportional to the concentration of the analyte, detected using a fluorometer. |
| **Raman spectroscopy** | Raman spectroscopy uses laser-induced scattering of light to analyze molecular vibrations. It identifies and distinguishes substances based on their unique spectral fingerprints. |
| **Spatially offset Raman spectroscopy (SORS)** | SORS uses Raman spectroscopy with varying laser offsets to penetrate and analyze subsurface layers of materials and is useful in situations where surface analysis alone may be insufficient. |
| **Surface enhanced Raman spectroscopy (SERS)** | SERS enhances Raman signals by using nanostructured surfaces which enables detection of low-concentration analytes as the Raman scattering of molecules is enhanced by several orders of magnitude. |
| **High-throughput trace-explosives detector** | A high-throughput trace-explosives detector detects minute traces of explosive materials. The sampler uses compressed air to detach particles from the surface of the target and a pre-concentrator to separate these particles from the airflow. The separated particles are vaporised by heating and analysed by the mass spectrometer. |
