# Supplementary material for: Where are you hiding the pangolins? screening tools to detect illicit contraband at international borders and their adaptability for illegal wildlife trafficking
Source: PLoS One. 2024 Apr 3;19(4):e0299152. doi: 10.1371/journal.pone.0299152 (PMC10990205; doi:10.1371/journal.pone.0299152)
Supplement: S8 Table — Detection tools described in the literature which primarily utilise acoustic or other methods. (DOCX) [file pone.0299152.s009.docx]

**Table S8. Acoustic and other detection tools.** Detection tools described in the literature which primarily utilise acoustic or other methods.

| **Inspection system** | **Description** |
| --- | --- |
| **Cantilever enhanced photoacoustic spectroscopy and quantum cascade laser (EC-QCL)** | EC-QCL uses photoacoustic detection to detect trace amounts of substances. Light directed into a sample excites molecules, raising energy levels, heating and pressurizing the surrounding gas. Modulated light creates sound waves, detected by a microphone and converted to an electrical signal. |
| **Acoustic/ultrasonic imaging** | Acoustic/ultrasonic imaging uses emits high-frequency sound waves and analyzes their reflections to create detailed images of hidden objects. Respiration can be detected through measuring the time-varying phase shift of the returning acoustic wave. Alternatively, reflections produced by discontinuities in containers which represent hidden compartments may be detected. |
| **Smart containers** | Smart containers are equipped with sensors and communication technology to continuously monitor cargo conditions. They detect GPS positioning and irregularities such as unauthorized access or changes in temperature and relay real-time data to authorities. Various different sensors may be equipped in the container. |
