# Supplementary material for: Where are you hiding the pangolins? screening tools to detect illicit contraband at international borders and their adaptability for illegal wildlife trafficking
Source: PLoS One. 2024 Apr 3;19(4):e0299152. doi: 10.1371/journal.pone.0299152 (PMC10990205; doi:10.1371/journal.pone.0299152)
Supplement: Attachment — Submitted filename: moloneyrev.pdf [file pone.0299152.s010.pdf]

Reviewer's Report:

Screening Tools to Detect Illicit Contraband at International Borders and Their Adaptability for Illegal Wildlife Trafficking: A Scoping Review

PLOS ONE manuscript #PONE-D-23-23299

1. There is too much detail on methods that are inapplicable to wildlife screening. Those methods need to be removed from the manuscript.

2. Most statements about the potential application of a method to wildlife detection are too vague to be useful. These descriptions need to be enlarged to include details as to exactly what modifications would be needed before a method could be used to detect wildlife in luggage or in shipping containers. For example, lines 461-464 are a good start but need much more detail.

3. There is little discussion of the challenges of applying a particular method to detecting wildlife within a particular setting such as airport passenger screening versus shipping container screening.

4. The manuscript's title in misleading. Actually, there is little in the way of a literature review. Most of the manuscript contains very brief descriptions of screening methods for detecting narcotics, explosives, or firearms. Similar descriptions can easily be found elsewhere. Hence, these descriptions need to be considerably abbreviated or, in many cases, removed entirely.
